# Liquid Resistive Switching Devices with Printable Electrodes

**DOI:** 10.3390/mi16080863

**Published:** 2025-07-26

**Authors:** Viet Cuong Nguyen

**Affiliations:** Institute of Advanced Technology, Vietnam Academy of Science and Technology, 1 Mac Dinh Chi, Ho Chi Minh City 70072, Vietnam; nvcuong@hcmip.vast.vn

**Keywords:** resistive switching devices, memristors, solution processes

## Abstract

In this work, research on liquid-based resistive switching devices is carried out, using bottom printable electrodes fabricated from Silver (Ag) paste and silver nitrate (AgNO_3_) solution. The self-crossing I-V curves are observed and repeatedly shown by applying 100 sweep cycles, demonstrating repeatability and stability. This liquid device can be refreshed by adding extra droplets of AgNO_3_ so that self-crossing I-V hysteresis with up to 493 dual sweeps can be obtained. The ability to be refreshed by supplying a new liquid solution demonstrates an advantage of liquid-based memristive devices, in comparison to their solid counterparts, where the switching layer is fixed after fabrication. The switching mechanism is attributed to Ag migration in the liquid, which narrows the gap between electrodes, giving rise to the observed phenomenon. The devices further show some synaptic properties including excitatory post-synaptic current (EPSC) and potentiation-depression, presenting opportunities to utilize the devices in mimicking some functions of biological neurons. The simplicity and cost-effectiveness of these devices may advance research into fluidic memristors, in which devices with versatile forms and shapes could be fabricated.

## 1. Introduction

Resistive memory devices are two-terminal devices, consisting of electrodes sandwiching an insulator [1]. This simple structure has brought many advantages, including a cost-effective and simple fabrication process. Therefore, they have played important roles as next-generation memory devices. Furthermore, the two-terminal structure also resembles synapses in a biological brain, in which pre-synaptic neurons are connected with post-synaptic neurons through synapses. As a result, resistive memory has been researched extensively in recent years as the basis for obtaining memristors which perform neuromorphic functions and possess novel characteristics [2,3].

Memristors are devices demonstrating pinched, self-crossing hysteresis current-voltage loops in a two-terminal device structure, comprising metal-switching layer-metal. The switching layers can comprise many different material systems, including oxides [4], polymers [5], and solution-processed materials [6]. Some works have used point-contact devices to study memristive devices in biomaterials [7] and oxides [8]; there are also some research works on fabricating memristive devices using printing techniques [9]. Another vibrant research field is on 2D materials-based memristive devices [10,11]. The 2D materials, such as Graphene, can be used as bottom electrodes for conformable devices on elastomers [12]; furthermore, composites of 2D materials and polymers were also utilized for demonstrating high-performance memristors on flexible substrates [13]. Additionally, by combining 2D materials’ exotic optical properties with a resistive switching layer, optoelectronic memristive devices could be realized [4]. Typically, the switching layer in memristive devices is a solid electrolyte, permitting ionic transport; thus, the mechanism could be attributed to the migration of oxygen vacancies and active ionic species like Ag [14]. The migration of these ions from the anode to the cathode will form metallic filaments, reducing the overall resistance of the device [14]. When a different voltage, in terms of magnitude or polarity, is applied, the filaments are broken or their cross-section is reduced due to Joule heating and electromigration, which leads to a resistance change in the device. The size and shape of these filaments can play pivotal roles in tuning the changes in the current with respect to the applied voltages, which is critical for realizing “plasticity” in brain functions where synaptic strength is increased or decreased depending on external stimuli [15]. The increase in synaptic strength is called potentiation, while the decrease is known as depression. Another interesting feature of memristive devices with Silver electrodes is the short retention time of the low-resistance state (LRS), when the compliance current (C.C) is set at a low level (typically 10^−6^ or 10^−5^ A) [16]. This feature is termed short-term memory and threshold switching, and it is proposed for applications involving mimicking nociceptors [17] or reservoir computing [18]. The mechanism of this feature is proposed by considering thin filaments with a small cross-section at the LRS, which was switched on with a low C.C; due to surface self-diffusion to minimize energy, this thin conducting filament can rupture, and many nanoclusters or nanospheres of Ag could be observed in the insulator matrix [19]. The self-diffusion is driven by the gradient of surface atomic vacancy concentration, which leads to clustering to lower the surface energy [20]. Although memristive devices with solid switching layers demonstrated many novel characteristics and high performance, their fabrication, which involves multiple-layer deposition techniques and expensive vacuum systems, could be challenging and costly.

Apart from solid switching layers, liquid solutions have also caught researcher’s attention owing to their cost-effectiveness and versatility. Additionally, since memristive devices are operated by ionic transport, a liquid could be promising as a switching layer because of its high ion mobility. Relaxation or “fading” characteristics of synapses can be realized conveniently in a liquid due to the diffusion of ions. Considering these advantages, some synaptic transistors with liquid gating have been studied [21]. In addition, memristive effects in two-terminal fluidic devices have also been demonstrated recently; the dynamics and migration of ions in these devices are the main mechanism [22,23,24]. However, there is still room for better understanding. In this work, we prepare resistive switching devices in the two-terminal form, using AgNO_3_ solution dropped on the bottom printable electrodes fabricated from Ag paste. The simple structure and process offer an opportunity to study and realize cost-effective memristive devices without vacuum deposition systems and expensive thin film equipment, thereby advancing the research toward all-printed memristive systems in any shapes and configurations [25]. Furthermore, with many recent exotic Silver-polymer composites [26] and stretchable metals [27], this research may pave the way for realizing innovative device concepts where novel integration with other fluidic sensing layers is feasible [28].

## 2. Materials and Methods

The bottom Ag electrodes were fabricated by depositing Ag paste on Polyimide substrates. They were then heated on a hot plate kept at a temperature above 100 °C for typically 3 min and 35 s until the solid layers were formed. After curing on the hot plate, the Ag electrodes were then polished by a metal plate until they were shiny. The electrical conduction of these electrodes was ensured by checking with a hand-held multimeter; typically, the resistance of the silver bottom electrodes measured by the multimeter was 1.3 Ω. For the switching layer, AgNO_3_ was dissolved in Deionized water (DI) at different concentrations, namely 0.05 M, 0.1 M, and 0.5 M. An AgNO_3_ liquid droplet of 3.5 µL was dropped onto Ag electrodes by a micropipette to make the switching layer. To complete the setup, a commercially available Gold (Au) tip was inserted into the droplet; this setup was also utilized in studying liquid-gated synaptic transistors [21]. The distance between the Au tip and the Ag electrode was about 0.5 mm. The Au tip was always fixed mechanically by double-sided tape.

Electrical measurements were performed on a Source Measure Unit, Keithley 4200. The bottom electrodes were grounded, and bias was applied to the top electrode for all the measurements. The compliance for I-V measurements was 10^−3^ A. The electrical measurement setup is given in Figure S1; all the measurements were carried out in this configuration unless noted separately. The silver deposited on the Au tips was observed by Scanning Electron Microscopes (SEM), and the element analysis was carried out with Energy Dispersive X-ray Spectroscopy (EDS).

## 3. Results and Discussion

**Figure 1:**
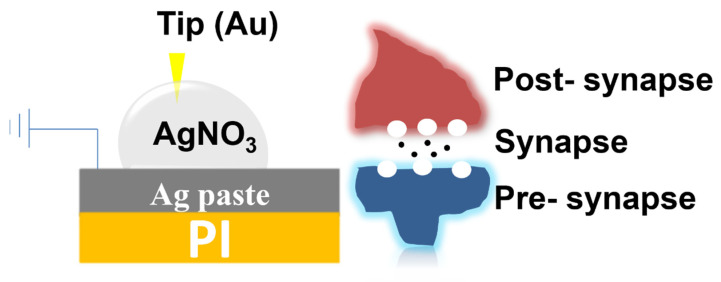
The schematic device structure of the liquid resistive switching devices.

Figure 1 shows the measurement setup and device structure. The device has a metal/liquid droplet/metal structure, similar to two-terminal resistive memory devices. The bottom Ag electrode is grounded and supplies Ag ions, while voltages are applied to the inert Au tip. The Ag electrodes play the role of pre-synaptic neurons while the Au tip is the post-synaptic one.

**Figure 2:**
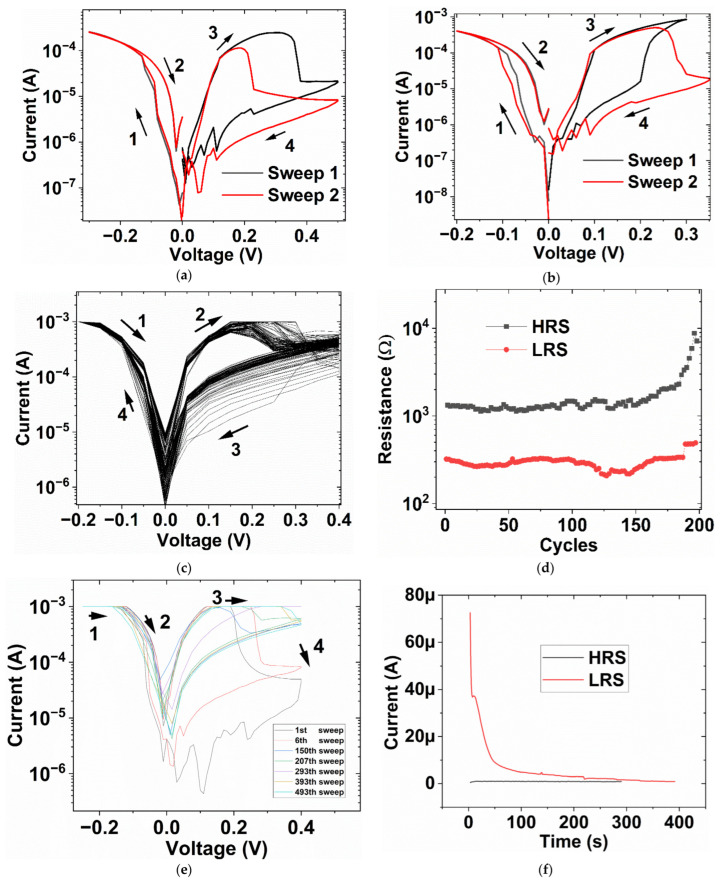
Current-Voltage characteristics of the liquid resistive switching devices (**a**) for 0.05 M [AgNO_3_] (**b**) 0.1 M (**c**) 0.5 M (**d**) Resistance-Cycles for 0.5 M [AgNO_3_] (**e**) 493 I-V sweeps of the liquid resistive switching device with 0.5 M [AgNO_3_], a new droplet was supplied at the 293th sweep, and (**f**) Retention time of the liquid resistive switching device with 0.5 M [AgNO_3_].

Sweeping is performed to show memristive effects. For all the concentrations (0.05 M, 0.1 M, and 0.5 M), hysteresis I-V switching can be observed. The hysteresis loops are self-crossing, which is the fingerprint of the memristors [29]. The “set” process is observed at negative voltage, while “reset” is at the positive one, which is consistent with the theory of electrochemical metallization [30]. Typically, the set voltage is between −0.05 V and −0.1 V, and the reset is between 0.2 V and 0.3 V. Figure 2a–c shows the electrical measurement results and the direction of sweeping. The switching “set” voltage is much lower in comparison with the solid state version due to high ion mobility in the liquid [31]. In addition, the devices can be operated without “forming” because the Ag ions are already in the solution through AgNO_3_; therefore, electroforming to drive the Ag from active electrodes is not needed. The electrical current of the devices depends on the concentration of AgNO_3_. It can be observed that the low-resistance state (LRS) current at −0.2 V increases when the concentration of AgNO_3_ increases. When the concentration of AgNO_3_ drops from 0.5 M to 0.05 M, the LRS current at −0.2 V declines from 10^−3^ A to 1.5 × 10^−4^ A. This can be explained by the fact that when [AgNO_3_] is low, thinner filaments with a smaller cross-sectional area are formed between the top and bottom electrode; hence, the current at LRS is lower. We observed that the device with the concentration of 0.5 M can be swept many times, up to 100 times (200 times of programming set and reset), as in Figure 2c,d; more self-crossing I-V hysteresis of 0.5 M [AgNO_3_] devices from two different days can be found in Figure S2. In Figure 2d, HRS and LRS at 0.05 V is obtained from 100 dual I-V sweeps in Figure 2c. After a long operation duration, the liquid droplet can become dried up, and a new one is added so that sweep cycles can continue. In Figure 2e, a new droplet was supplied at the 293rd sweep cycle. The set-reset process in Ag-based devices is controlled by the formation and rupture of Ag filaments through migration and redox reactions. In the liquid state, due to high ion mobility, diffusion of Ag from filaments into the liquid is high, which could give rise to difficulties in maintaining the LRS [24]. Furthermore, the ability to “refresh” the liquid-based resistive switching devices, demonstrated by the possibility of supplying a new liquid solution, may offer exciting opportunities in realizing reconfigurable devices. Typically, in solid memristive devices, the switching layers are unalterable after the vacuum deposition; this fabrication process also requires capital-intensive investments. Figure 2f shows the retention time of LRS and HRS. It can be seen that the LRS current at 0.03 V decays with time and approaches the HRS at 0.03 V, after approximately 300 s. This decaying feature can be attributed to the diffusion of Ag particles from the filament to the surrounding liquid medium, which weakens the conducting filament.

**Figure 3:**
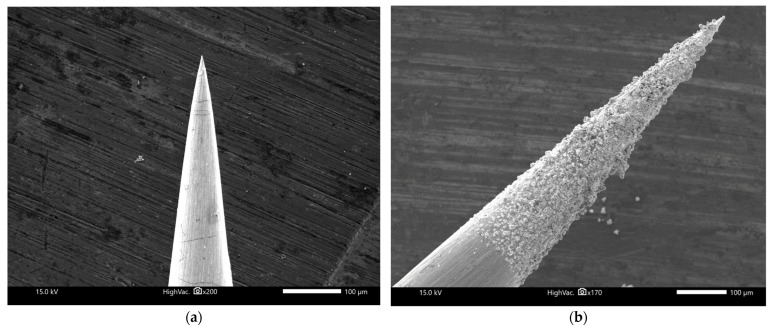
Scanning Electron Microscopy (SEM) image of (**a**) the pristine Au tip and (**b**) the tip after operation. The scale bar in (**a**,**b**) is 100 µm.

Ag migrating from the anode and nucleating at the cathode has been proposed as a mechanism for electrochemical metallization memory devices [30]. At the anode, the Ag electrode becomes oxidized into Ag^+^ ions, and these ions migrate toward the cathode, where they are reduced to Ag^0^. The filaments then grow in the form of dendrites from the cathode toward the anode, switching the resistance of the device from a High-Resistance State (HRS) to an LRS. Based on the electrochemical metallization mechanism, it is reasonable that the cathode in this switching process will be covered with islands or layers of Ag after many cycles of set-reset. Hence, after performing multiple I-V sweeps in devices using 0.1 M [AgNO_3_], the Au tip was rinsed thoroughly in pure water and transferred to the SEM chamber for characterization. Figure 3a shows the pristine Au tip; it is apparent that the tip is sharp without particles or particulates on the body as well as the tip’s end; the tip’s surface is smooth. In Figure 3b, a rough deposited layer is found on the tip’s end and body after I-V sweeps. The EDS spot analysis in Figure S3 of the supporting information shows that this layer is made predominantly of Ag, which supports the hypothesis above and confirms that electrochemical metallization can be used to explain the working mechanism of these liquid resistive switching devices. In order to observe the phenomenon from another angle, a lateral device was made as shown in Figure S4a. After I-V sweeps from −0.4 V to 0.7 V (Figure S5), the Ag dendrites could be observed with an optical microscope in the gap between the Ag electrode and the Au tip (Figures S4b,c and S6). This further corroborates the electrochemical metallization mechanism, and in fact, the electrochemical migration of Ag in an aqueous environment has been extensively reported in the literature [32].

**Figure 4:**
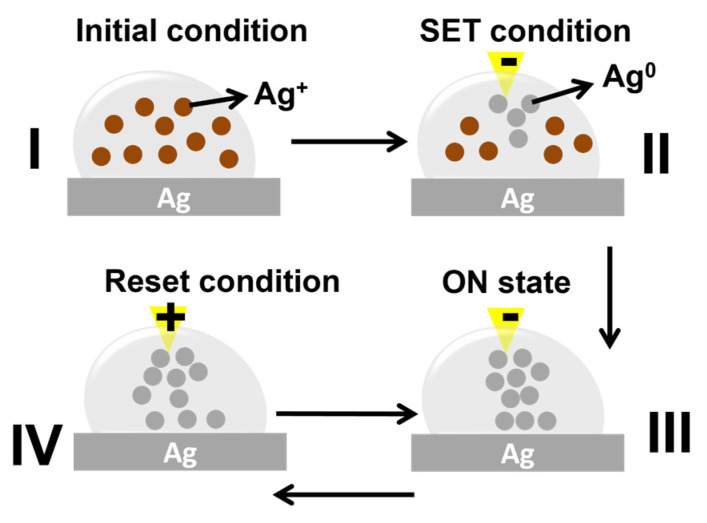
The proposed switching mechanism.

The mechanism of this memristive switching can be attributed to Ag filaments forming and rupturing between the electrodes. When negative voltage is applied to the Au tip (set process), Ag from the anode is oxidized into Ag^+^ and injected into the solution. The Ag^+^ ions in the solution are transported to the cathode (inert Au tip), where they receive electrons to become metal Ag^0^. The growth of Ag metallic dendrites from the cathode to the anode brings the state of the device to LRS (“ON” state). In this device, multiple filaments are expected [30]. When a positive voltage is applied to the Au tip (reset process), the filament is ruptured due to Joule heating and redox reactions at the filament’s end; the device’s state is brought into HRS (“OFF” state). The reset process occurs because the curvature of the filament is much higher than that of the planar Ag electrode [30]. In this reset process, the front-most Ag dendrite twig has a positive potential, which promotes the oxidation reaction, Ag → Ag^+^ + e^−^, and the deposition/ electrochemical reduction takes place at the planar Ag electrode. Since the filament tip has a tiny volume and high curvature, leading to a strong electric field concentration, faster oxidation takes place, and a significant portion of the filament is dissolved. Re-deposition of this dissolved section of the filament on the planar Ag electrode only advances the geometric front of this electrode to an inconsiderable extent toward the filament, which is dissolved rapidly. As a result, the gap between the filament and the planar Ag electrode widens, giving rise to a drop in electrical conduction [30]. The sweeping cycles that devices with 0.5 M [AgNO_3_] can sustain are larger than those of other devices with lower concentrations because the concentration of Ag^+^ ions is higher in the former (0.5 M) than in the latter (0.05 M and 0.1 M). The mechanism is summarized in Figure 4, and further schematic pictures of the “reset” process can be found in Figure S7.

**Figure 5:**
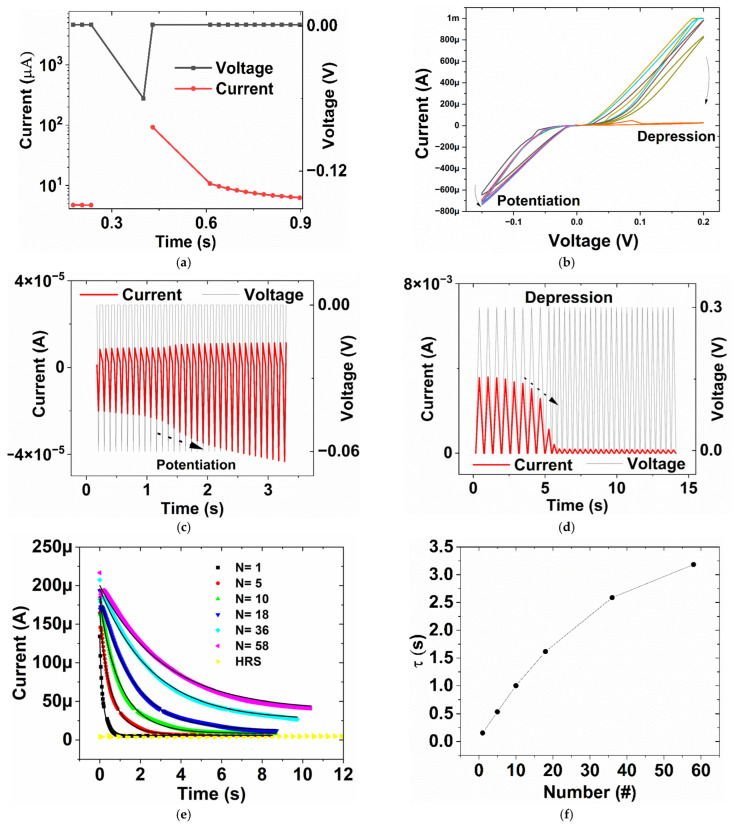
Synaptic functions of the liquid resistive switching devices (**a**) Excitatory Post-synaptic Current (EPSC) (**b**) Potentiation and depression of the devices’ current by consecutive negative voltage sweeps from 0 V to −0.15 V and consecutive positive voltage sweeps from 0 V to 0.2 V (**c**) Current response, showing potentiation, when saw-tooth spikes from 0 V→−0.06 V→0 V are supplied (**d**) Current response, showing depression, when saw-tooth spikes from 0 V→0.3 V→0 V are supplied (**e**) Current response at 0.03 V versus the numbers of electrical stimuli, solid lines are the fitted curves following the SEF model, and (**f**) The fitted relaxation time constant τ versus the numbers of stimuli.

Synaptic functions can be obtained in these liquid resistive switching devices with 0.5 M [AgNO_3_]. In Figure 5a, the device’s current at 0.001 V is initially high at about 92.8 × 10^−6^ A. After a negative spike of −0.06 V is applied to the device in the time scale of approximately 0.17 s, then this response current decays to 6.2 × 10^−6^ A. This decay of post-synaptic current is a characteristic of EPSC [33]. The mechanism of the decay is due to the diffusion of Ag from the filament into the liquid medium. The full current and voltage versus time of the EPSC can be found in Figure S8. In addition, the device also shows potentiation and depression characteristics when consecutive stimuli are applied. In Figure 5b, the measured currents are plotted as a function of applied voltages for five consecutive negative voltage sweeps from 0 to −0.15 V and five positive sweeps from 0 to 0.2 V. For negative sweeps, the current at −0.15 V increases when the number of sweeps increases, while for positive sweeps, the current at 0.2 V decreases. This demonstrates the potentiation and depression of the device’s output current, similar to the characteristics of synapses in biological systems [33,34]. Similarly, when saw-tooth voltage spikes are applied to the devices, we can observe typical potentiation and depression in the current response. In Figure 5c, 33 potentiating spikes from 0 V→−0.06 V→0 V are supplied, and the evoked output current at −0.06 V rises. For 33 depressing spikes from 0 V→0.3 V→0 V in Figure 5d, the current peak at 0.3 V falls with time [35,36].

Another intriguing feature of memristive devices is the ability to mimic memory loss and memory transition from the short-term memory (STM) to the long-term memory (LTM) [37,38,39,40]. The STM to LTM transition is related to the Spike Number Dependent Plasticity (SNDP) [40]. In Figure 5e below, the changes in current at 0.03 V with respect to time are shown, after different numbers of identical electrical stimuli (N) at −0.2 V have acted on the 0.5 M [AgNO_3_] device. The current at 0.03 V is recorded right after the train of −0.2 V stimuli. An electrical stimulus is a spike from 0 V→−0.2 V→0 V. Voltage and current responses versus time for different stimuli N are shown in Figure S9. When N = 1, the response current of the device decays rapidly, reaching the current value of the HRS measured at 0.03 V. However, when N increases, the decay becomes less severe and stabilizes when N reaches 58 stimuli. This shows the STM to LTM transition. In fact, the decay resembles “forgetting” in the biological brain [37], and can be modeled by the Stretched Exponential Function (SEF) [40,41]

(1) $$I=\left(I_{0}-I_{\infty }\right)e^{-{\left(\frac{t}{\tau }\right)}^{\beta }}+I_{\infty }$$

where I_∞_ is the final value of the decaying current, I_0_ is the initial current right after the train of stimuli, τ is the characteristic relaxation time constant, and β is the stretching index ranging between 0 and 1. Equation (1) indicates an abrupt drop when t < τ; as a result, decaying characteristics can be described by the relaxation time constant τ. The larger the time constant, the less severe the decaying current could be observed. In addition, this equation can also be used to describe memory loss in the human brain [38]. The SEF is used to fit the response current in Figure 5e, and the fitting curves are shown as the solid lines. The obtained relaxation time constant τ increases with the number of stimuli, as shown in Figure 5f below. The “multistore model” by Atkinson and Shiffrin suggests that STM can be developed into LTM by a process of rehearsal repetition [37], which is well mimicked in Figure 5e,f. The filamentary-based mechanism can explain this observed phenomenon well; when the number of stimuli rises, thicker filaments are formed, leading to a more stable conducting channel.

## 4. Conclusions

In this work, we demonstrate liquid resistive switching devices on printable electrodes fabricated from Ag paste. The device shows self-crossing I-V hysteresis loops for many sweeping cycles, indicating repeatability and stability. The mechanism of the switching is attributed to Ag migration from the inert electrode to the active electrode, which lowers the device’s resistance. Using SEM, Ag layers can be found on inert Au tips after electrical switching, which supports the electrochemical metallization mechanism. Some synaptic functions, such as EPSC and potentiation-depression, were also demonstrated in this liquid device. Using printable electrodes, the devices can be made without expensive vacuum processes, which may pave the way for future cost-effective memristive devices. In addition, printable electrodes could enable innovative device concepts in which memristive devices in any shape or form factor can be realized. Furthermore, the possibility of replenishing the liquid medium facilely can open up opportunities for realizing reconfigurable fluidic memristive devices and mimicking closely the biological synapses; in fact, the human body is a large liquid system, where body fluids are exchanged with the outside environment to maintain functions and remove waste products.

## Data Availability

The data presented in this study are available on request from the corresponding authors.

## Supplementary Materials

The following supporting information can be downloaded at: https://www.mdpi.com/article/10.3390/mi16080863/s1, Figure S1: The electrical measurement setup, Figure S2: The self-crossing I-V hysteresis of 0.5 M [AgNO_3_] devices obtained from two different days, Figure S3: EDS spectrum of the coated layer on the Au tip, Figure S4: A lateral liquid resistive switching device with [AgNO_3_] = 0.5 M, Figure S5: I-V hysteresis of the lateral device in Figure S4, Figure S6: Zoom-in of Figure S4c at the junction between the Ag electrode and the Au tip, Figure S7: The schematic picture of the “reset” process, Figure S8: The full current and voltage of EPSC current vs time, described in Figure 5a of the main text, Figure S9: Voltage profile and current response with respect to different numbers of stimuli. The identical stimuli at −0.2 V are applied at the beginning, followed by a constant “read” voltage at 0.03 V.

## Abbreviations

The following abbreviations are used in this manuscript:

LRS	Low-Resistance State
HRS	High-Resistance State
SEM	Scanning Electron Microscopy
EDS	Energy Dispersive X-ray Spectroscopy
EPSC	Excitatory Post-Synaptic Current
C.C	Compliance Current
STM	Short-Term Memory
LTM	Long-Term Memory
SEF	Stretched Exponential Function
