# Supplementary material for: Liquid Resistive Switching Devices with Printable Electrodes
Source: Micromachines (Basel). 2025 Jul 26;16(8):863. doi: 10.3390/mi16080863 (PMC12388810; doi:10.3390/mi16080863)
Supplement: Supplementary file 1 [file micromachines-16-00863-s001.zip › micromachines-3729688-supplementary.pdf]

Supporting information

Liquid resistive switching devices with printable electrodes

Viet Cuong Nguyen

Institute of advanced technology, Vietnam Academy of Science and Technology, 1 Mac Dinh Chi, Ho Chi Minh City, Viet Nam

Email: nvcuong@hcmip.vast.vn

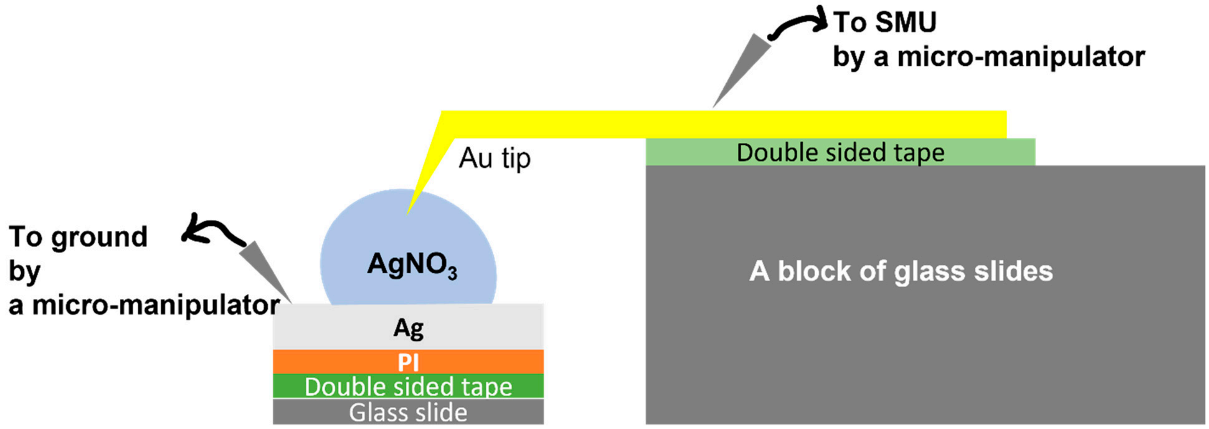

Figure S1. The electrical measurement set-up.

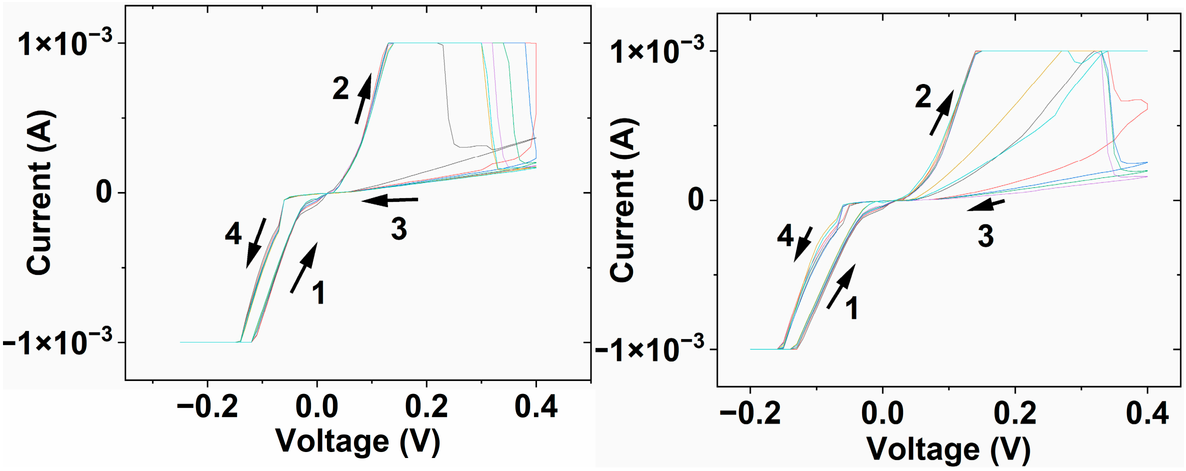

Figure S2. The self-crossing I-V hysteresis of 0.5 M [$AgNO_3$] devices obtained from two different days.

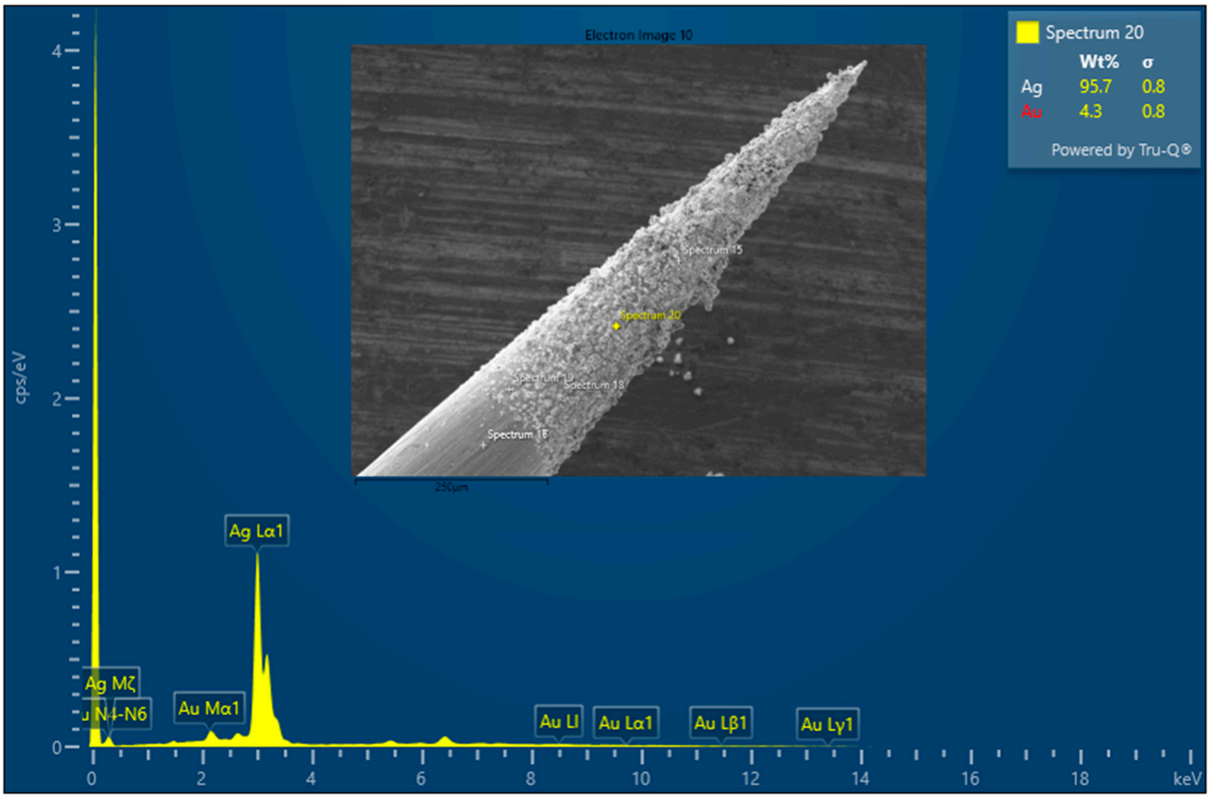

Figure S3. EDS spectrum of the coated layer on the Au tip. The yellow dot in the inset indicates the spot where EDS is performed.

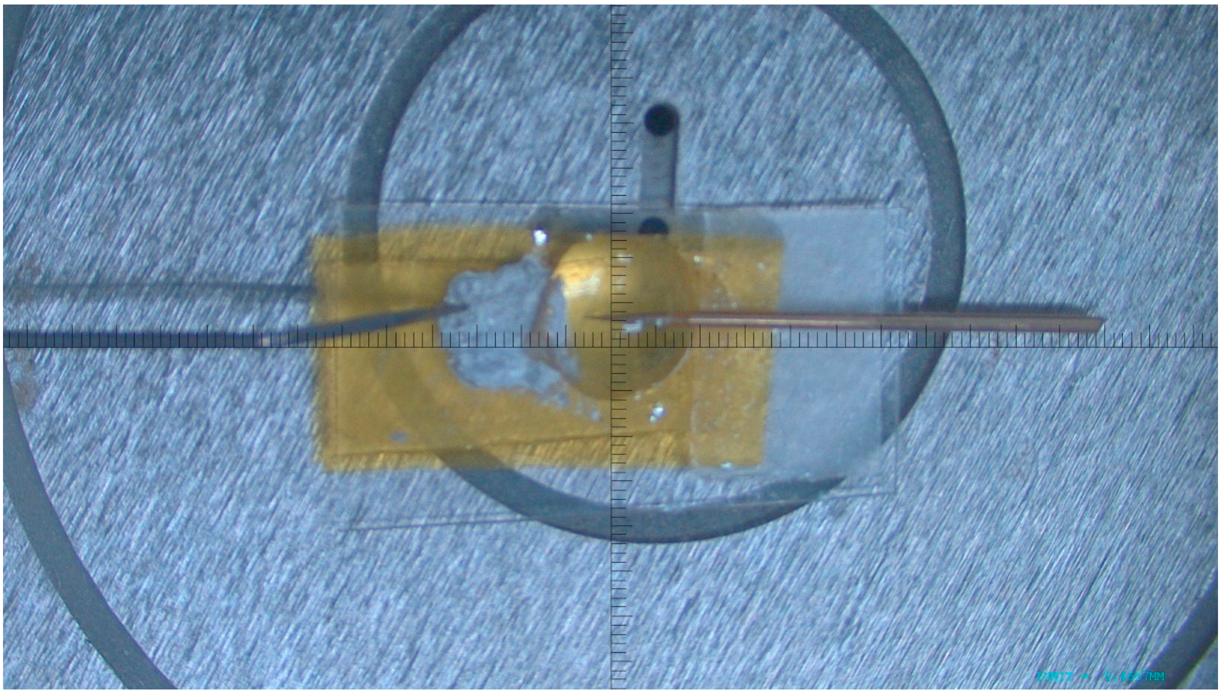

(a)

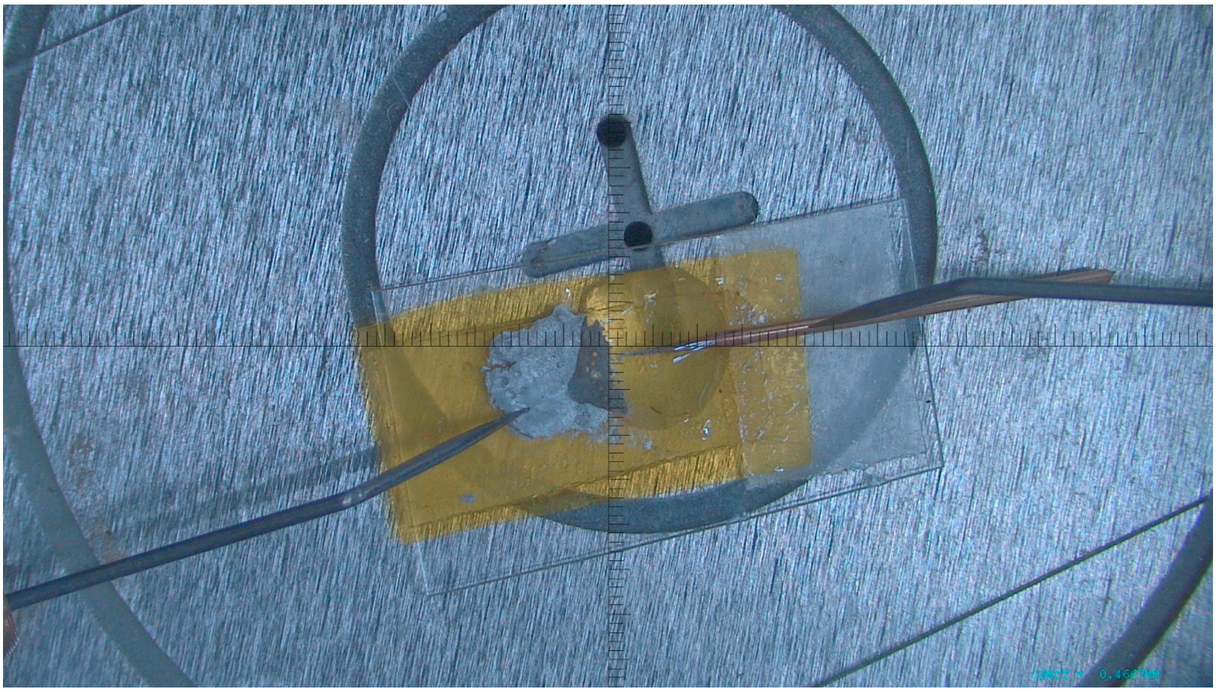

(b)

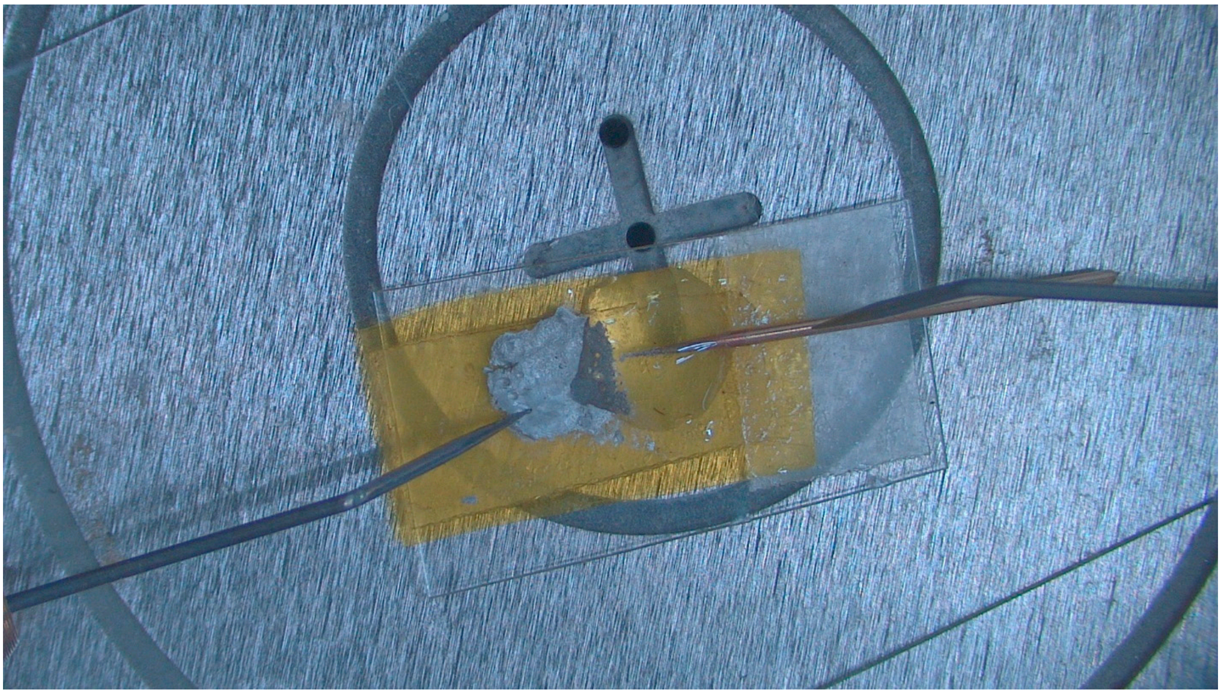

(c)

Figure S4. A lateral liquid resistive switching device with [$AgNO_3$] = 0.5 M. (a) Before applying voltage sweeps. (b) and (c) After applying voltage sweeps. (b) With a scale bar and (c) without a scale bar. 1 unit in the scale bar equals 0.4667 mm.

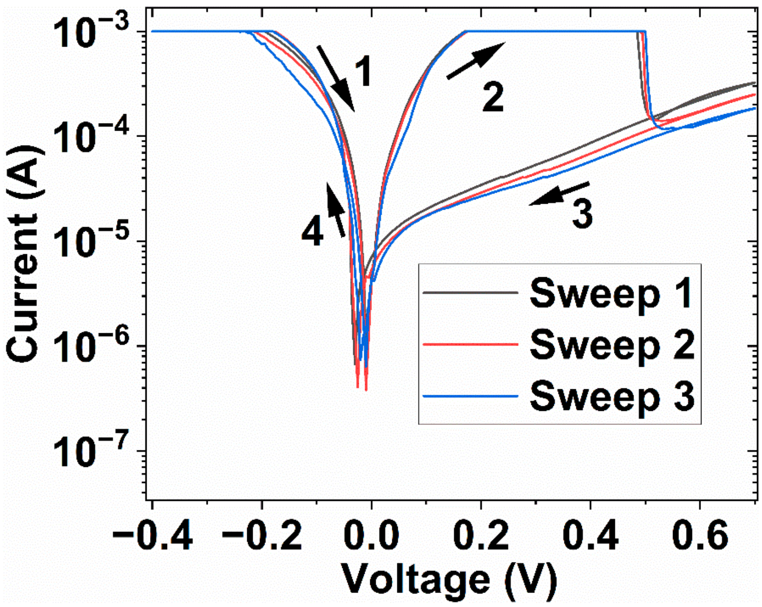

Figure S5. I-V hysteresis of the lateral device in Figure S4.

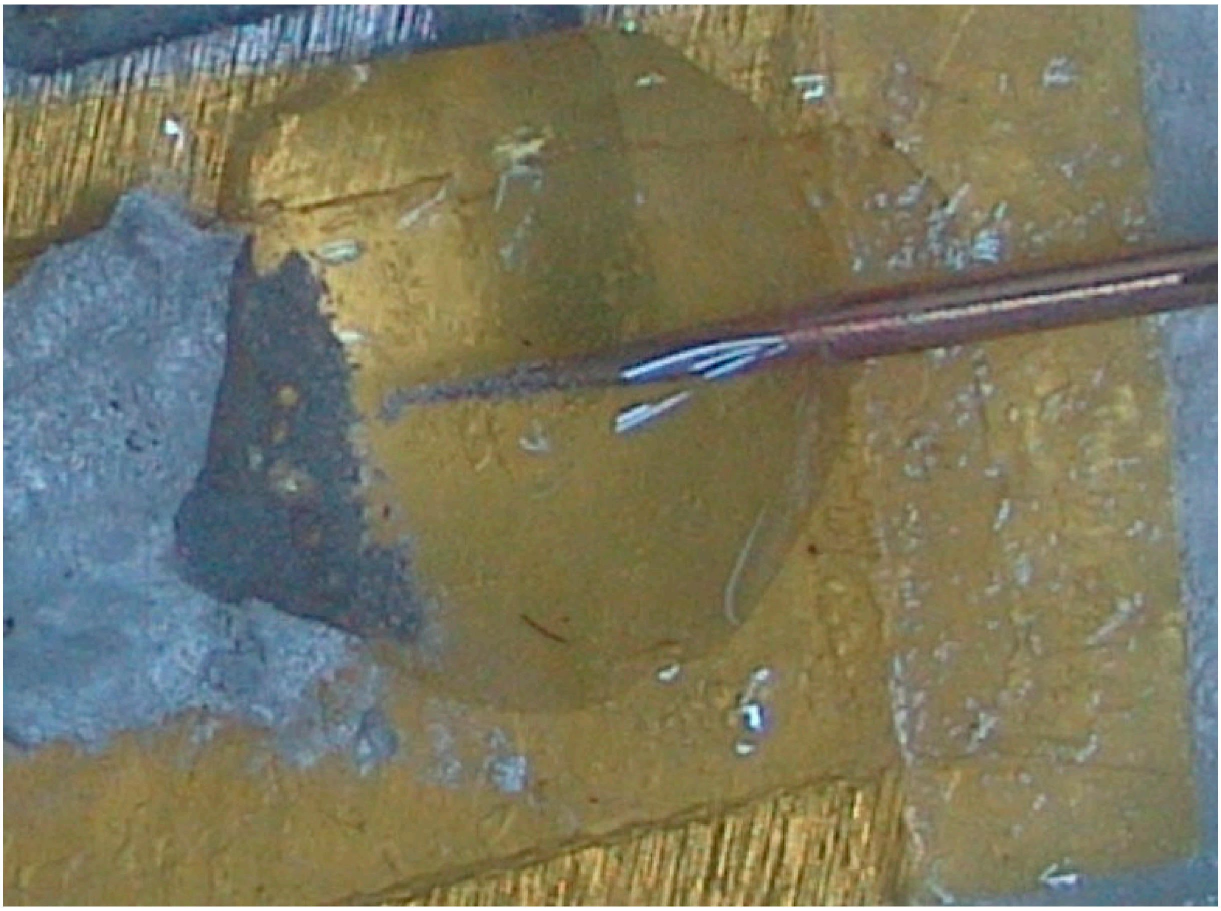

Figure S6. Zoom-in of Figure S4c at the junction between the Ag electrode and the Au tip.

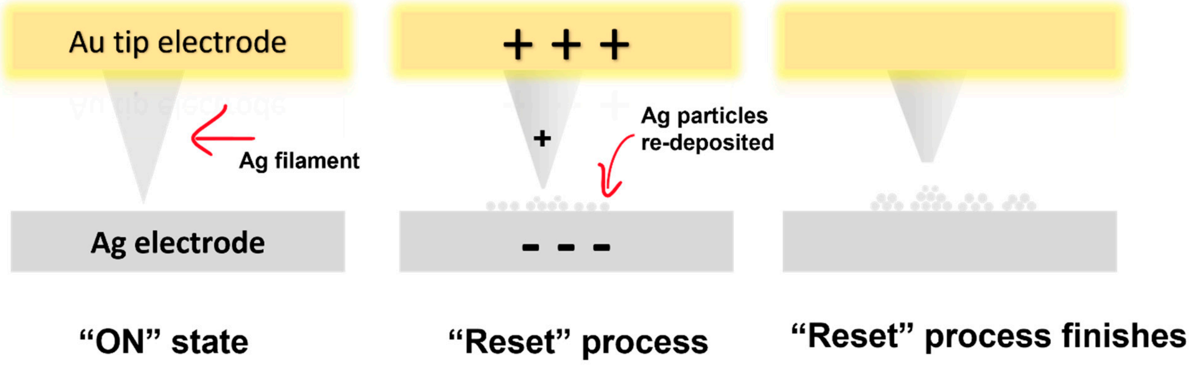

Figure S7. The schematic picture of the "reset" process.

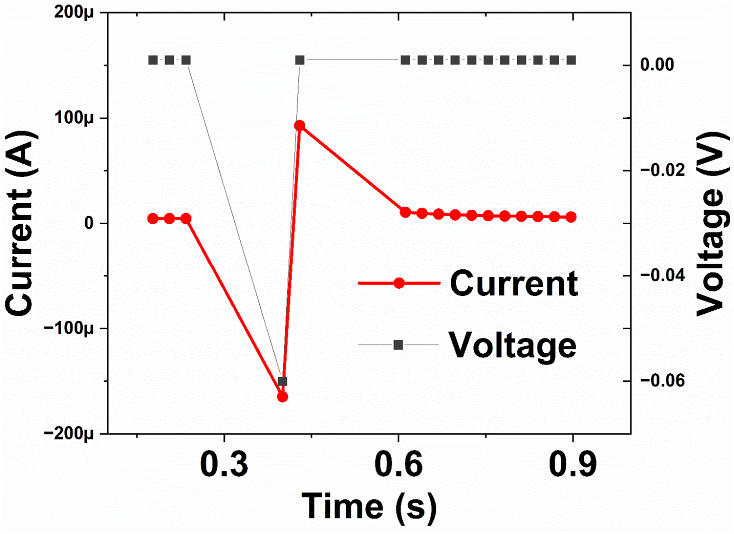

Figure S8. The full current and voltage of EPSC current vs time, described in Figure 5a of the main text.

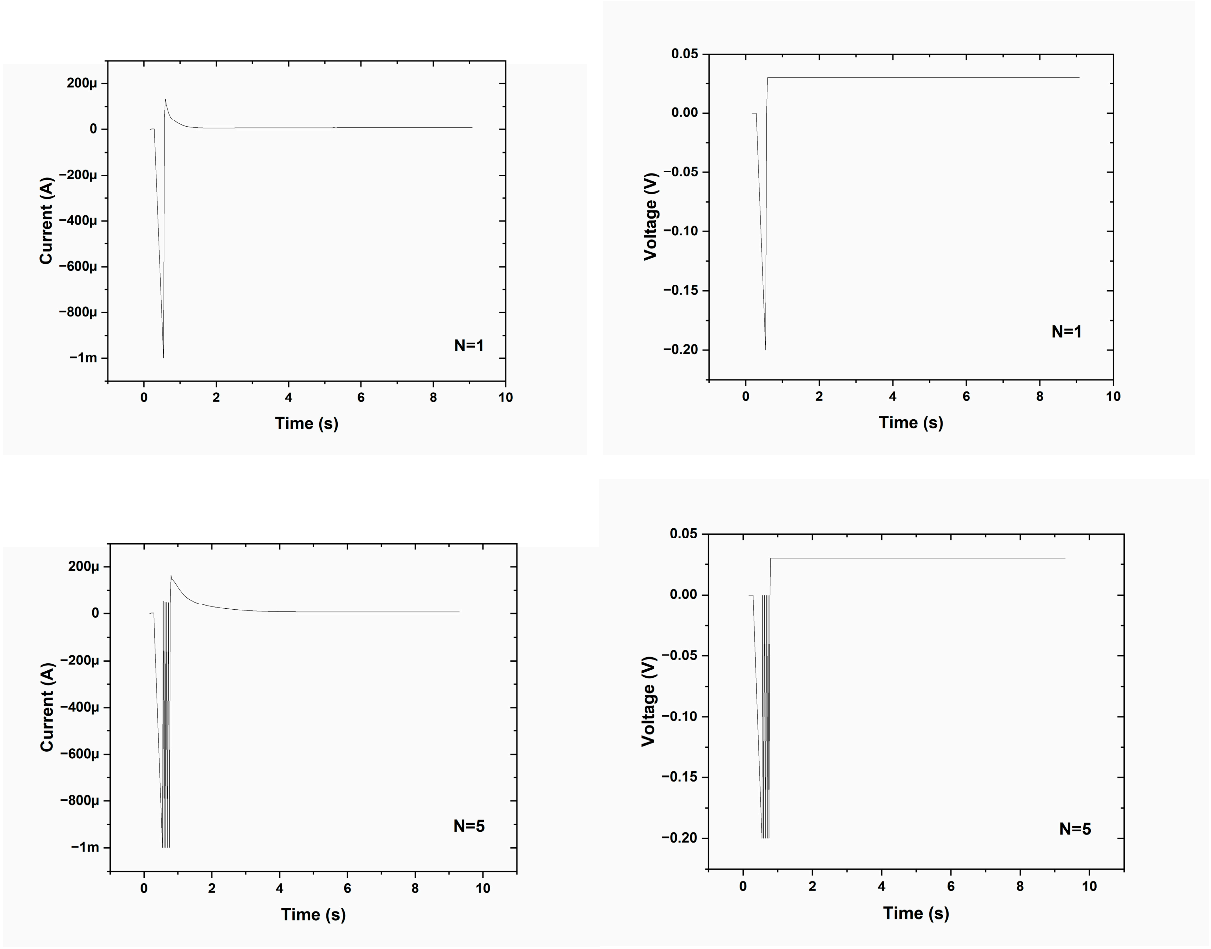

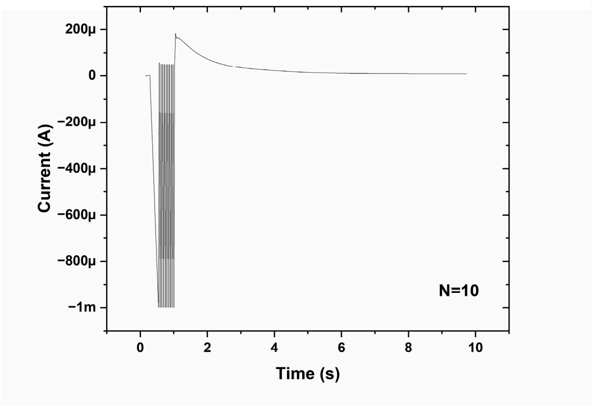
Current (A)
200μ
0
−200μ
−400μ
−600μ
−800μ
−1m
0
2
4
6
8
10
Time (s)
N=10

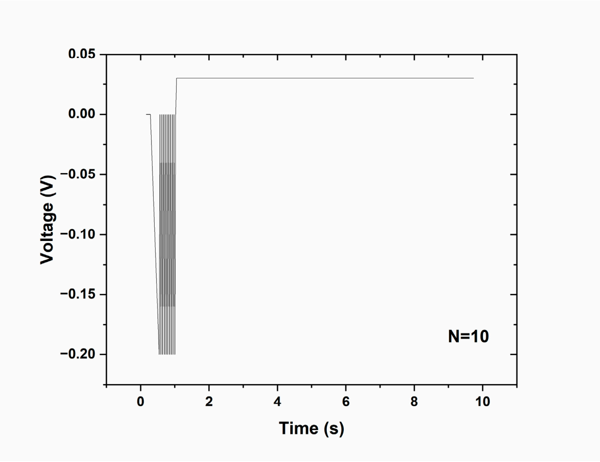
Voltage (V)
0.05
0.00
−0.05
−0.10
−0.15
−0.20
0
2
4
6
8
10
Time (s)
N=10

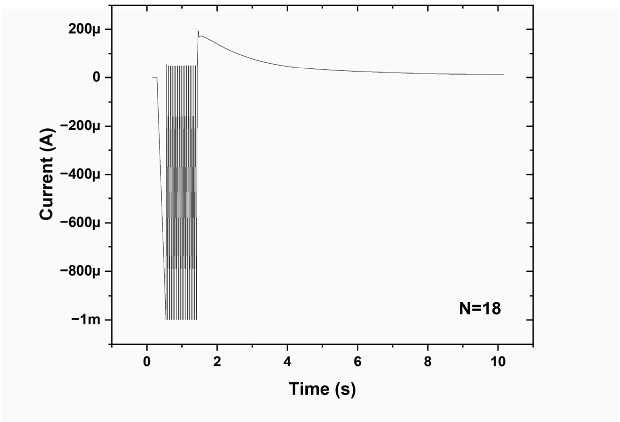
Current (A)
200μ
0
−200μ
−400μ
−600μ
−800μ
−1m
0
2
4
6
8
10
Time (s)
N=18

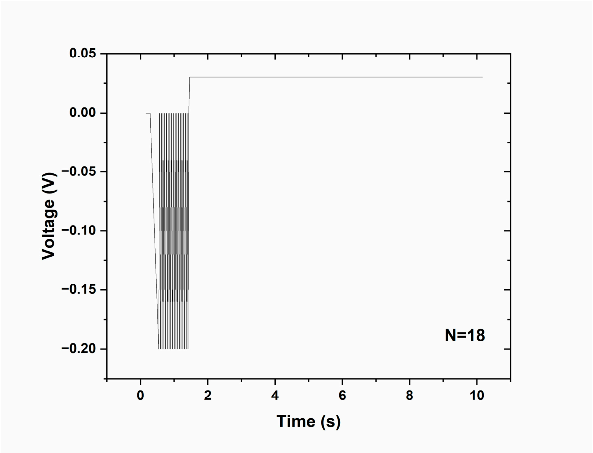
Voltage (V)
0.05
0.00
−0.05
−0.10
−0.15
−0.20
0
2
4
6
8
10
Time (s)
N=18

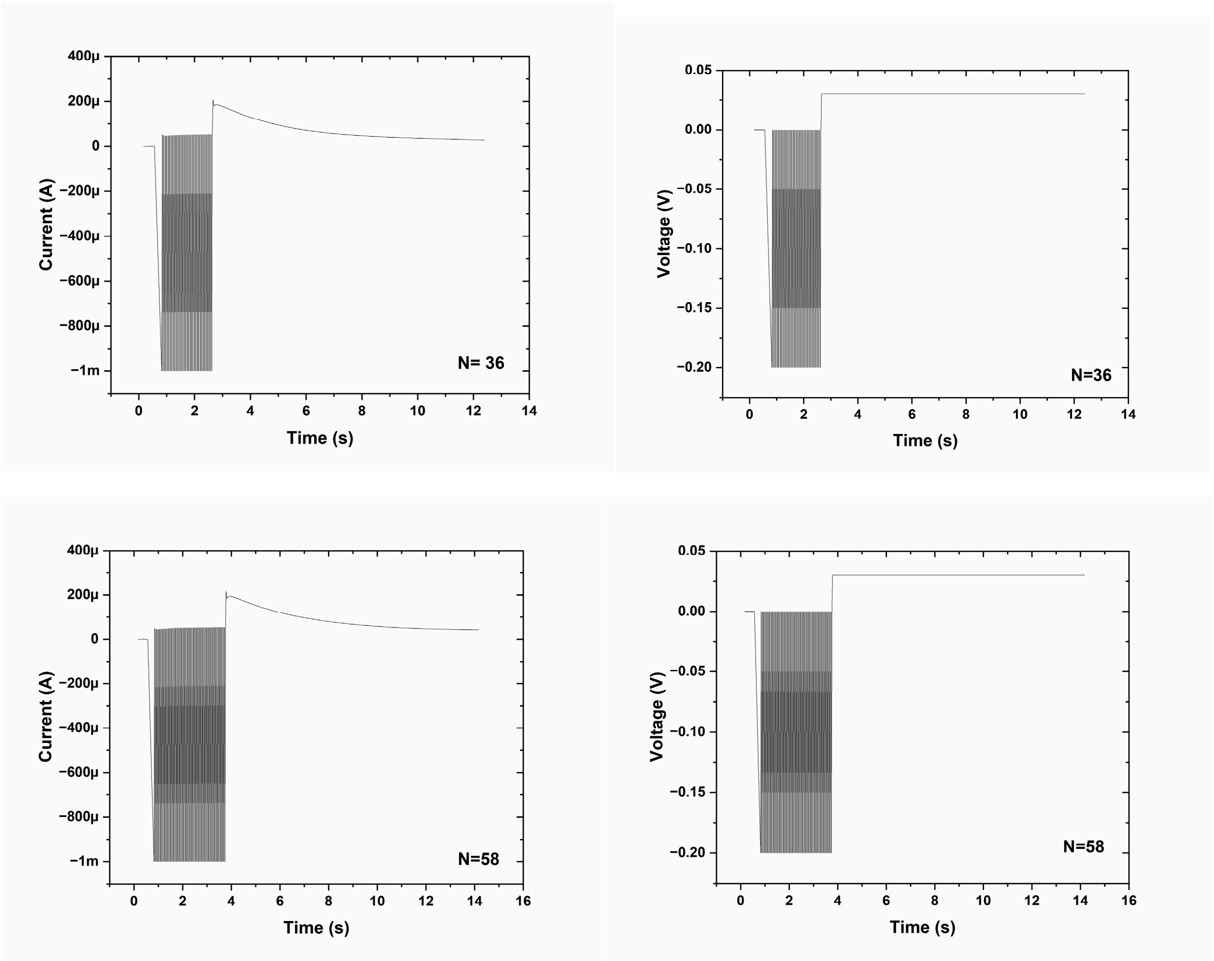

Figure S9. Voltage profile and current response with respect to different numbers of stimuli. The identical stimuli at -0.2 V are applied at the beginning, followed by a constant "read" voltage at 0.03 V.
